# Impact of Fabric Properties on Textile Pressure Sensors Performance

**DOI:** 10.3390/s19214686

**Published:** 2019-10-28

**Authors:** Luca Possanzini, Marta Tessarolo, Laura Mazzocchetti, Enrico Gianfranco Campari, Beatrice Fraboni

**Affiliations:** 1 Department of Physics and Astronomy, University of Bologna, Viale Berti Pichat 6/2, 40127 Bologna, Italy; luca.possanzini2@unibo.it (L.P.); enrico.campari@unibo.it (E.G.C.); beatrice.fraboni@unibo.it (B.F.); 2 Department of Industrial Chemistry, University of Bologna, Via Risorgimento 4, 40136 Bologna, Italy; laura.mazzocchetti@unibo.it

**Keywords:** textile pressure sensor, e-textile, dynamic mode, PEDOT:PSS

## Abstract

In recent years, wearable technologies have attracted great attention in physical and chemical sensing applications. Wearable pressure sensors with high sensitivity in low pressure range (<10 kPa) allow touch detection for human-computer interaction and the development of artificial hands for handling objects. Conversely, pressure sensors that perform in a high pressure range (up to 100 kPa), can be used to monitor the foot pressure distribution, the hand stress during movements of heavy weights or to evaluate the cyclist’s pressure pattern on a bicycle saddle. Recently, we developed a fully textile pressure sensor based on a conductive polymer, with simple fabrication and scalable features. In this paper, we intend to provide an extensive description on how the mechanical properties of several fabrics and different piezoresistive ink formulation may have an impact in the sensor’s response during a dynamic operation mode. These results highlight the complexity of the system due to the presence of various parameters such as the fabric used, the conductive polymer solution, the operation mode and the desired pressure range. Furthermore, this work can lead to a protocol for new improvements and optimizations useful for adapting textile pressure sensors to a large variety of applications.

## 1. Introduction

In recent years, the field of wearable technology has attracted great attention and different kinds of sensors have been developed. The advent of this new technology is changing the way people interface with the external world. Wearable electronic sensors are growing very popular in several fields such as medical [1], healthcare [2,3,4], wellness [5], entertainment [6,7] and safety [8]. The most requested applications deal with wearable activity trackers that provide a powerful and detailed self-monitoring as well as an opportunity to directly control personal habits and behaviors. Such devices are usually based on inorganic electronics chips and are considered wearable because they are in contact with the body, typically the wrist. However, the most promising completely wearable and imperceptible sensors are those fabricated directly on fabric, that is, fully textile physical or chemical sensors. Fabrics, with their comfort and fitting features, are the best platforms and substrate onto which to directly realize a wearable sensor device and, when provided with sensing properties, are named *Smart Textiles*. They can be made sensitive to various external stimuli, both physical and chemical [9], such as the changes in force [10,11], pressure [12,13], deformation [14,15], temperature [16] or the concentration of specific compounds in body fluids [17], like ion chloride [18] or sweat quantity [19]. Furthermore, it is possible to directly functionalize fabrics with organic polymers in order to realize fully textile electrocardiogram electrodes for the detection of biopotentials [20,21]. Such devices represent a valid tool for the early diagnosis of diseases and monitoring. Wearable textile devices require that the electronic and data communication systems have to be flexible and lightweight to allow their integrability into the textiles themselves. This paper is focused on fully textile wearable pressure sensors. Many efforts have been devoted to developing sport garments and accessories to reduce injuries [22], increase the athlete’s performance [23], comfort, or improve safety in the work place. Depending on the desired area of application, the pressure sensors have to be tuned to work in low or high pressure ranges with a reasonable sensitivity. Those with high sensitivity in the low pressure range (<10 kPa) allow touch detection in human-computer interaction or to develop an artificial hand for handling objects. In the same way, sensors working in a high pressure range (>10 kPa) can be used to monitoring the body pressure distribution in standing or sitting position. However, even if the linear response range and high sensitivity are the two main factors that describe a pressure sensor performance, it is necessary to consider its response related to a dynamic pressure change when it targets a real-life application. Very scarce and random details are reported in the literature on standard characterization procedures that would allow a comparison of the different proposed sensors’ performances and actual fields of applicability.

Different examples of wearable pressure sensors are reported showing novel and simple configurations with high sensitivity, but most reported results have been obtained in a single step pressure measurement or in a very slow dynamic mode, that is, conditions that are far from the real-life applications. For instance, polyethylene combined with Carbon sheet has been applied to a glove and show good responses in a range between 1 kPa and 70 kPa. In this case, the sensor performance has been evaluated with a delay from 30 s to 180 s to reach rest conditions after each measure [24]. Another example is a piezoresistive sensor based on a modified graphite polyurethane that shows a slow dynamic range of almost 10 kPa/min [25]. On the contrary, good results have been obtained with a capacitive sensors [26], based on a graphene sponge with a wide response range (0–50 kPa), high sensibility of 0.96 kPa^−1^ and a good dynamic response demonstrated in a fast mode of 75 kPa/min. We recently developed a pressure sensor based on a conductive polymer printed on cotton fabric [22]. The poly (3,4-ethylenedioxythiophene): poly (4-styrenesulfonate) (PEDOT:PSS) is screen printed on the fabric and then two conductive threads are sewed to complete the electrical connections. This fabrication process is easy and low cost, with potential scalability for large-scale production. Even if the pressure sensor is rather simple on its own, its interaction with the textile nature of the substrate yields a complex system. The description of its working principle should take into account these challenging issues. We have recently reported an interpretation identifying three different contributions to textile pressure sensors performance [27]. At a macroscopic level the fabric properties, in terms of thickness, wettability and mechanical properties, are the most important factors that determine the pressure sensor performance. Going down to the microscopic level, the coating of the fibers affects the sensor response and sensitivity; finally, we identified a third contribution at the nano-level which influences the linear response range and the pressure saturation.

In this paper, we further deepen the investigation and the understanding of the mechanism controlling the performance of textile pressure sensors and present a detailed study of how the fabric type, weaving and structure influences their performance in dynamic operation mode. Four fabrics, with different material composition, thickness, sewing method and layered structure have been characterized and used as substrate for the pressure sensors. We performed electrical and mechanical tests (creep-recovery and stress-strain) both on plain fabrics and on the textile pressure sensors we fabricated onto each fabric, evaluating the sensor performance in dynamic mode operation in order to measure how the mechanical features of the fabrics affect this operation regime. We calculated and evaluated the sensor linear response in a wide pressure range. We used the same geometry and structure for all pressure sensors and we tested two different conducting polymer formulations—one based on pristine PEDOT:PSS and one obtained adding the ethylene glycol (EG)-like second dopant. We demonstrate that sensors based on the second formulation (more electrically conductive) show higher sensitivity, wider linear operation range but lower reliability compared to those realized with the first formulation (pristine solution). These results highlight the complexity of the textile pressure sensor system due to the active role of multiple parameters (i.e., the fabric composition, structure, the polymer formulation, the targeted pressure range, etc.) and suggest a protocol to optimize and tune textile pressure sensors to different types of application.

## 2. Materials and Methods

### 2.1. Materials

**Figure 1:**
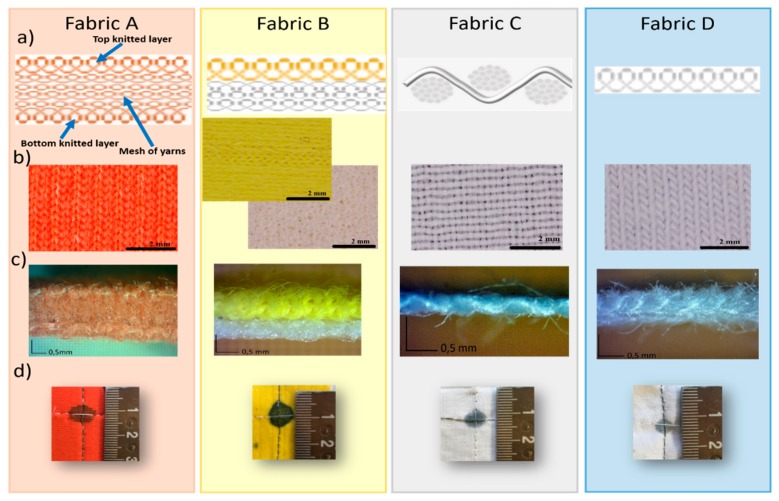
(**a**) Schematic representation of the fabric weaving structure; optical images of top (**b**) and lateral (**c**) view. (**d**) Appearance of one pressure sensor spot.

Four different fabrics, with different material composition, weave arrangement, layered structure and thickness were taken into account, considering both knitted and woven fabrics (Figure 1). The woven fabrics are all commercially available and are made by interlacing in a regular order two sets of yarns so that they cross each other perpendicularly. The lengthwise yarns are called warp while the crosswise yarns are called weft. Normally, they interlace each other at right angles. On the contrary, the knitted fabrics are made by interlocking a series of loops composed by one or more yarns, with every single row of loops locked into the previous row. The lengthwise loops are called wales and the crosswise loops are called courses.

Fabric A consist of three layers with the outside ones composed by one single sheet of knitted structure. The embedded part is a system of yarns disposed perpendicularly with respect to the top and bottom sides. Fabric B is composed of two layers of knitted yarns distinguished by the different threads color. Fabric C, instead, is based on woven fabric. Finally, we also consider an elastic knitted textile, fabric D, whereby the special lengthwise stretchability is given by the particular knitting way of the yarns.

The conductive polymer poly (3,4-ethylenedioxythiophene): poly (4-styrenesulfonate (PEDOT:PSS), Clevious P, is purchased by Hearaeus. Two formulations of conductive ink have been studied—formulation Clevios P based on pristine PEDOT:PSS and formulation Clevios P+EG based on PEDOT:PSS with 10% *v*/*v* of ethylene glycol (supplied by Sigma-Aldrich).

The pressure sensors were prepared by drop casting the ink directly onto the fabric using a pipette. Fabrics A and B were drop cast with 25 μL in both sides, while the fabrics C and D were treated with 5 μL on one side. The conductive ink was dried at room temperature. A commercial 2 ply stainless steel conductive thread (resistivity of ~10 ohms/m) has been sewn with lockstitch by the sewing machine Elna eXa 320 in the final pressure sensor configuration as described elsewhere [27].

### 2.2. Fabric Chemical Characterization

Fourier transform-infrared (FT-IR) spectra were recorded on a Brucker Alpha Platinum-Attenuated Total Reflectance (ATR) spectrophotometer equipped with ATR Diamond window (32 scans, 4 cm^−1^ resolution).

Differential Scanning Calorimetry (DSC) measurements were carried out on a TA Instruments DSC Q2000 apparatus equipped with Refrigerated Cooling System RCS90, heating twice 3–5 mg samples in aluminum pans from −50 °C to 30 °C at 10 °C/min, with intermediate cooling run carried out at −10 °C/min. The instrument is calibrated with Indium standard.

### 2.3. Fabric Mechanical Characterization

TA Instrument Dynamic Mechanical Analysis (DMA) Q800 has been used in compression mode to characterize the mechanical properties of fabrics and sensors. The maximum force provided by DMA is 18 N, resulting in a normal pressure up to 100 kPa. In a creep recovery test, a constant pressure of 100 kPa was applied to the fabrics for 1 min and, after release, the strain recovery and thickness were recorded for three minutes. Stress-strain response has been evaluated with ten stress cycles from 0 kPa to 100 kPa at a speed of 50 N/min (~4.6 kPa/s).

### 2.4. Sensor Characterization

Pressure sensors performance has been studied using the DMA in compression mode together with the source meter Keithley 2400 as a read-out electronics. The same conditions described above for fabric characterization were used for the stress-strain tests. The compression top clamp has an area of 1.77 cm^2^ (larger than the sensor active area) in order to provide an equal pressure distribution throughout the sensor. The source meter unit supplies a constant current of 1 μA, with a sampling time of 200 ms, meanwhile it measures the sensor resistance variations. This configuration allows monitoring the pressure, the thickness and the electrical behavior of the textile pressure sensors.

## 3. Results and Discussion

### 3.1. Characterization of Fabrics

**Figure 2:**
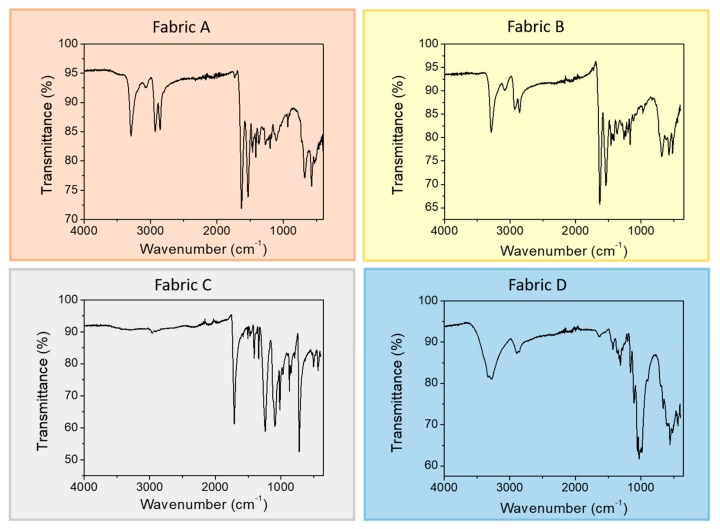
Fourier transform infrared (FT-IR) pattern for the four fabric studied.

All fabrics were analyzed in all of their components. Fabric A, an orange soft material, always displays a FT-IR pattern typical of nylon fibers (Figure 2), a synthetic material that is widely used for technical textiles [28]. While absorptions recorded in ATR mode account for a typical polyamide (i.e., nylon), with the 3293 cm^−1^ absorption ascribed to free N-H stretching in solid state, 1630 cm^−1^ band that is typical of carbonyl stretching (amide I band), and 1532 cm^−1^ signal ascribed to NH bending vibration (amide II band) [29] and 1, infrared spectroscopy is not helpful in identifying the exact composition of the polymer. Hence the thermal behavior of the fabric was analyzed in DSC, and the resulting thermogram in the second heating scan (Figure S1) displays two main events, both endothermic: the highest temperature signal centered at 252 °C that is typical of melting of the Nylon 6,6, crystal phase, the latter being a polyamide widely used in textile industry. The low temperature peak, which is persistent and not removed with the first heating scan intended for cancelling the thermal history of the sample, can instead be attributed to the melting of some waxy element applied as sizing coating to the fibers during the weaving processing.

Though fabric B displays a clear difference, at least in the coloring (white and yellow), of the two layers, when analyzed with the ATR-IR technique the sample displays exactly the same spectrum irrespective of the analyzed layer. As displayed in Figure 2, where the yellow layer spectrum is reported, the absorption pattern strongly resembles the previously discussed spectrum of fabric A, which was attributed to a polyamide fiber, identified as mainly Nylon 6,6. DSC analysis of fabric B, however, reveals a different thermal behavior than fabric A (Figure S1), with the presence of a high-T endothermic peak that is similar in position to the one attributed to the melting of Nylon 6,6, while a new endothermic event, positioned at a slightly lower T, is now present. The position of such a peak can be coherent with the melting of other commonly used polyamides, such as, among others, Nylon 6. Moreover, fabric B thermogram shows just the hint of a signal in the same position of the low-T endotherm observed for fabric A.

The fabric C ATR-IR spectrum displays a significantly different profile than the previously discussed ones (Figure 2), with features ascribed to a polyester filament. Indeed, the spectrum displays the major peaks associated with the structure of polyethylene terephthalate (PET) with the terephthalic acid ester carbonyl stretching at 1712 cm^−1^, the asymmetric C–C–O and the O–C–C stretching at 1240 and 1091 cm^−1^, respectively, and the C–H wagging vibrations from the aromatic structures at 722 cm^−1^ [30]. The DSC thermogram confirms this hypothesis, displaying a single endothermic event centered at 252 °C (Figure S1), that can be associated with the melting of PET crustal phase. Such a polyester is once again well renowned for its use in textiles and garments.

The last investigated sample, fabric D, shows an infrared absorption that is different from all the previously analyzed fibers (Figure 2), with the lack of any C = O feature, some prominent broad OH stretching band in the region 3500–3000 cm^−1^ and C-O-C signals in the 1150–1075 cm^−1^ region that are reminiscent of a cellulosic structure. Cellulosic fibers are the most relevant natural fibers used in textiles, both as cotton and flax fibers, suggesting that fabric D might be a cotton fabric. Cotton is not expected to provide any significant thermal signal in DSC analysis, since cellulosic fibers thermally degrade before being able to undergo any thermal transition. However, DSC second heating scan displays a low-T signal (Figure S1), in a position similar to that previously detected in fabric A and B. In order to assess whether this is an additive, as previously hypothesized, fabric D was washed in water with soap, as a common garment. DSC analysis of the washed sample confirm the disappearance of the peak (Figure S2), supporting the hypothesis that this might be a processing coadjutant used in the weaving process.

### 3.2. Mechanical Characterization of Fabrics

**Figure 3:**
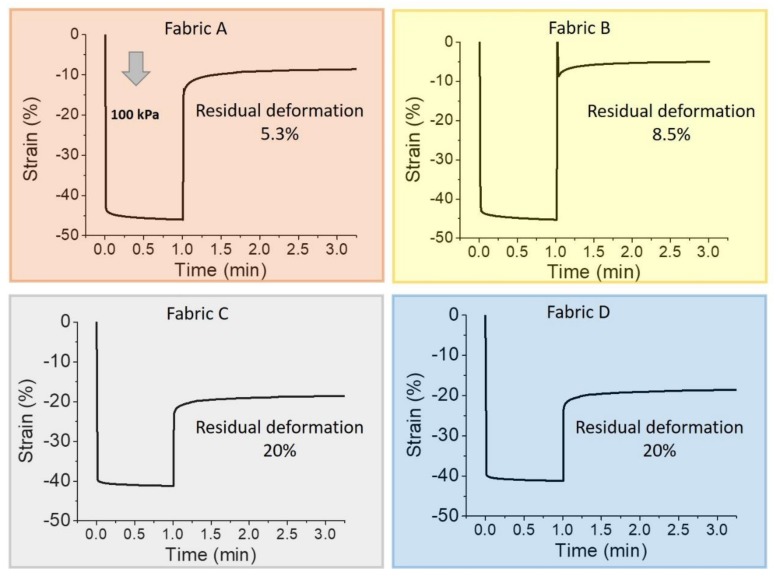
Mechanical characterization of the 4 different tested fabrics under creep recovery test: the arrow indicates the applied pressure (100 kPa for 1 min); the recovery time is 3 min.

We focused the structural analysis on the vertical properties of the fabrics because this is usually the relevant direction of the textile pressure sensors in real-life applications. To investigate and compare their properties we first performed a creep-recovery test. We selected the highest pressure used in this study (100 kPa) and we applied it for 1 min. The recovery curve was recorded for 3 min. This test provides information about the vertical elastic properties of each fabric and an influence on the textile pressure sensor performance is expected. Figure 3 shows the creep-recovery trends and the relevant parameters are summarized in Table 1.

The thickness of fabrics A and B are considerably different compared to the thickness of fabrics C and D. Strain is therefore the most appropriate quantity to be used in order to compare the creep-recovery test results. The maximum strain value reached by every fabric under the 100 kPa pressure was quite the same (~45%). Observing the residual deformation, fabric A and fabric B have good elastic properties with high recovery up to 5.3% and 8.5%, respectively. On the other hand, fabric C results are less elastic and its residual deformation is 20%. Comparting it with fabric D, we observed a similar behavior with the same recovery response. These results underline the fact that the elastic consistency of fabric D (the elastic one) mainly concern the horizontal stretching and does not affect the vertical mechanical properties.

**Figure 4:**
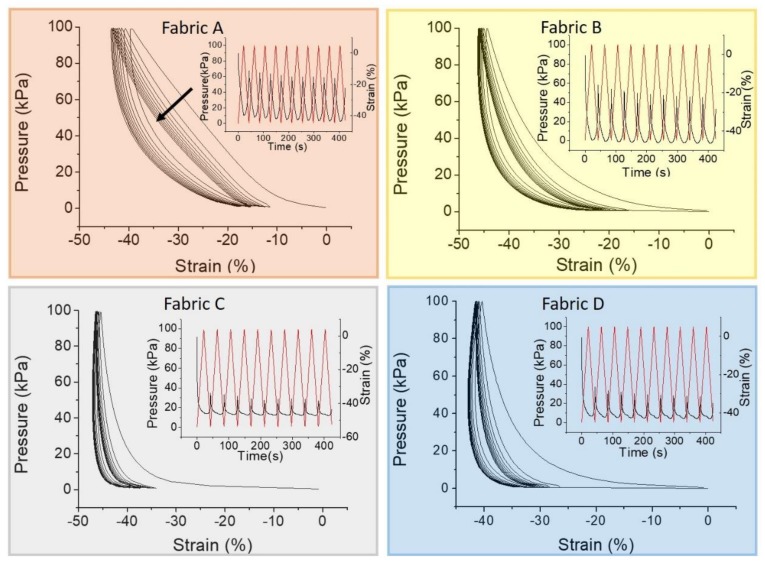
Stress-strain curve of fabrics: the arrow indicates the curve shift direction from the first to the tenth cycle. Inset: pressure (black line) and strain (red line) as a function of time for each fabric.

Even though the creep recovery test gives relevant information about the fabric elastic properties, it does not represent their actual behavior in the textile pressure sensors during a dynamic operation mode. For this reason, we performed a cycle of compression and decompression stress-strain test. The stress-strain curves, in which the stress value represents the pressure, are reported in Figure 4. The fabrics show a substantial variation on the stress-strain curve after the first cycle. This is in agreement with the previous results, in which a complete recovery has not been observed for any fabric (Figure 3). The stress-strain test confirms that fabric A has good elastic properties, indicated by the linearity of the compression curve for each cycle. However, it does not have enough time to complete the recovery and after each cycle the strain curve does not overlap the previous one.

On the other hand, fabric B does not show proper elastic properties and the compression curve is not linear. However, apart for the first one, the strain curves are increasingly overlapping in subsequent cycles. Fabrics C and D do not show a linear compression curve. After the first cycle, both fabrics remain compressed but each strain curve almost overlaps that of the previous cycle. To better evaluate this aspect, the insets of Figure 4 show the strain against time (right axis) while cycled pressure is applied (left axis). In this representation, it is possible to observe the dynamic strain range (the difference between the maximum and the minimum strain value in a cycle) and the symmetries of the strain curve during compression and decompression. Ruling out the first cycle, in fabric A the strain value ranges from −10% to −40% for the second cycle, and from −13% to −43% for the tenth cycle, resulting in a large dynamic strain range of ~30%. As observed before, fabric B seems to be less elastic than fabric A but the strain values are more reproducible and range from −15% to −40% with a dynamic strain range of ~25%. Since the symmetry between compression and decompression curves, we can consider both fabrics provided with reasonable elastic features. The thinner fabrics behave differently, underlining the fact that the structure/geometry characteristics of textile can influence the response. They show a greater thickness variation after the first cycle and a lasting compression during the others. They have a lower dynamic strain range of about 10%. The strain values range from −35% to −45% for fabric C and from −40% to −30% for fabric D. Moreover, the compression and decompression regions of the strain curve are not symmetric and the smallest thickness does not correspond with the highest stress.

### 3.3. Pressure Sensor Performance in Dynamic Mode

We fabricated textile pressure sensors with each fabric, following the procedure described in the experimental method. The textile sensors, based on conductive polymer PEDOT:PSS, consist of two conductive stainless steel threads that are in contact with each other through a fabric coated with such a polymer. Increasing the applied pressure, the thickness of the fabric decreases, the piezoresistivity of conductive polymer changes and the whole pressure sensor resistance decreases (conductance increase) [22,27].

**Figure 5:**
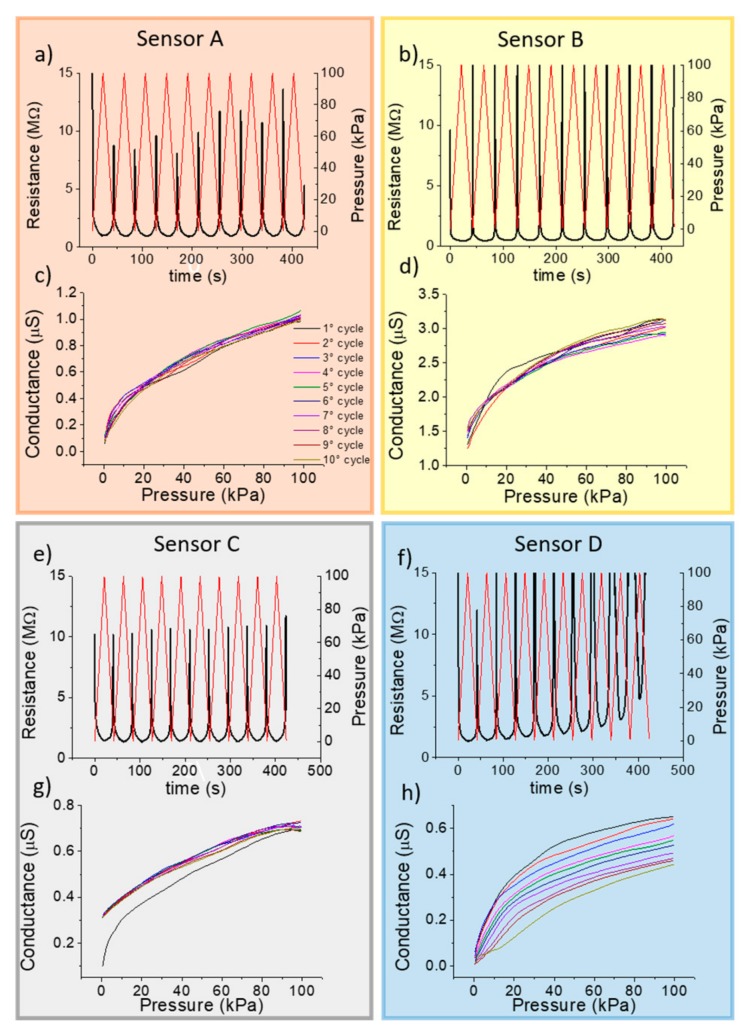
Comparison of the four fabrics. (**a**,**b**,**e**,**f**) Resistance sensor response in dynamic mode for 10 cycles with an applied pressure from 0 to 100 kPa with a frequency of 5 kPa/s. (**c**,**d**,**g**,**h**) Conductance trend during compression for every single cycle.

From now on, we will call sensor A the textile pressure sensor fabricated using fabric A, sensor B the one realized with fabric B and so on. Due to the differences in thickness, we increased the volume of the conductive polymer in the drop casting deposition in order to achieve a proper and comparable vertical coating also in thicker fabrics. For this reason, sensors A and B have a sensitive area (A = (51.6 ± 0.69) mm^2^; B = (48.5 ± 0.6) mm^2^) larger than the area of pressure sensors C and D (C = (33.2 ± 0.5) mm^2^; D = (18.5 ± 0.4) mm^2^). The upper graph of each section in Figure 5 shows the resistance response (black, on the left) during the application of 10 pressure cycles (red, on the right). The performance of the textile pressure sensor has been evaluated in the same condition as the stress-strain test. The resistance curve is inversely proportional to the applied pressure because, when the pressure increases, the device resistance decreases. A linear response of the electrical properties with the applied external pressure is more suitable for real-life applications. Thus, for our textile pressure sensor, it is useful to evaluate the conductance instead of the resistance. Plotting the conductance, a linear shape in some regions can be achieved. Figure 5c,d,g,h show the conductance trend under compression for each pressure cycle.

Sensors A and C, even if the fabrics used are substantially different, have a stable and reproducible response. Sensor C shows a strong variation after the first cycle because woven polyester fabric needs a first compression cycle to stabilize the vertical structure of its fibers. This behavior is also confirmed from the stress-strain test for this fabric. The conductance response for sensor B presents a small spread through 10 cycles that increases in the high pressure range. Sensor D, with a single layer of knitted cotton fabric, is the least stable because an increase in the resistance with the number of cycles is observed. 

As already observed, the change of the conductive polymer formulations affects both its electrical and its piezoresistive properties and we assessed the effects of varying the conductive polymer formulation on the performance of textile pressure sensors. The consequences in the textile pressure sensors can be identified as a different performance and a different pressure operating ranges. We added to the PEDOT:PSS formulation 10% *v/v* of ethylene glycol which is a well know second dopant that enhance the conductivity of the organic compounds [31]. The cyclic conductance of the sensor response with the addition of EG is reported in Figure S3.

**Figure 6:**
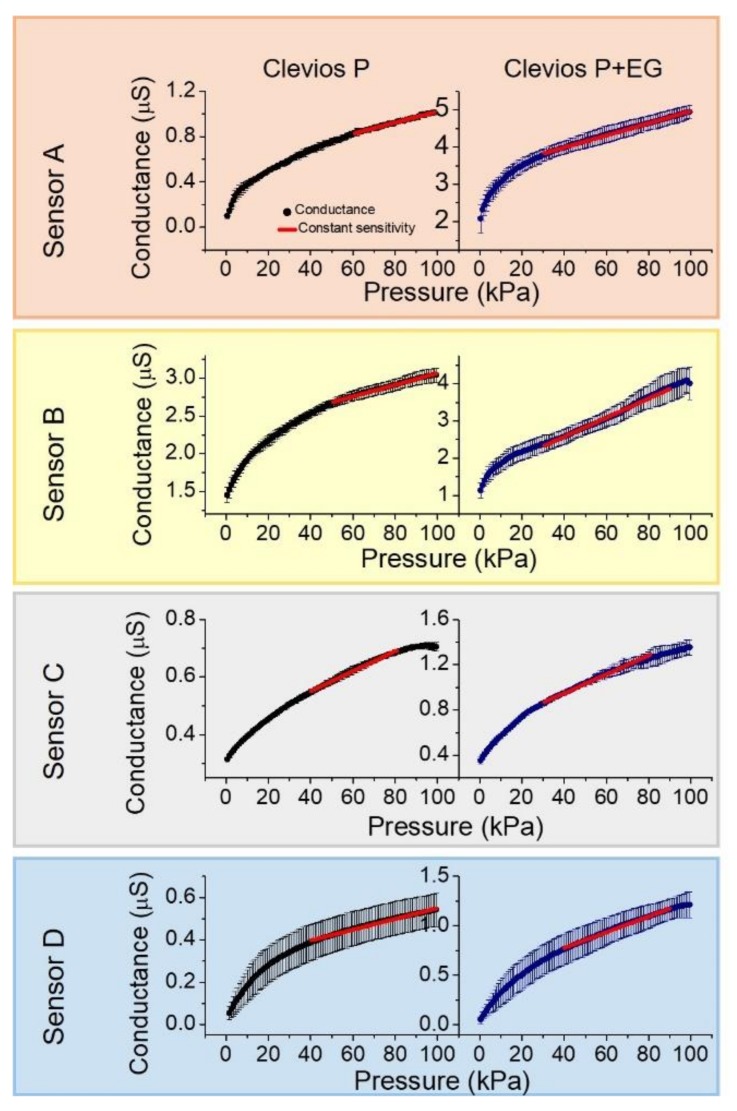
Average trend over ten cycles of the conductance response during compression stress for both formulations.

Figure 6 shows the average values of conductivity at each cycle with and without the presence of EG for textile pressure sensors fabricated with comparable geometry and structure onto the 4 different fabrics here tested. The linear working range, sensitivity and reliability are indeed affected by EG, the conductivity enhancement agent. The sensors based on the more conductive formulation have a greater sensitivity regardless of the fabric and a wider linear working range. However, the sensors with ethylene glycol present a less reliable behavior, as highlighted by larger standard deviation values. In these dynamic tests, we have not observed any saturation in the conductance as a function of pressure up to values of more than 70 kPa. This behavior is usually observed in a static characterization of the sensors realized with a more conductive ink in which the large number of conductive points between the PEDOT-rich zone that covers every single fiber enabling the achievement of a maximum conductance value before the application of the highest pressure.

The linear conductance response (G) is modeled as $G\;=\;S\cdot P+G_{off}$, with S the sensitivity expressed in nS/kPa. G_off_ is the intercept value that is not zero because a preload force of 0.05 N is applied. Figure S4 shows the derivative of the conductance for every fabric in both formulations. The derivative represents the point sensitivity values and the constant region of these curves identify the sensors linear operation range. For a better estimation of this working range, the statistical $\chi^{2}$-test was taken into account as descibed in the Supplementary Information.

The most relevant parameters to interpret the sensors performance are summarized in Table 2: (i) the conductance G_0_ at the lowest applied pressure of 0.5 kPa; (ii) the conductance G_100_ when the pressure is 100 kPa and the sensitivity values calculated with the weighted least mean squares method. The sensitivity of a sensor is an essential parameter that describes how the estimated output varies when there is a variation in the input values. In this case, the output variable is the conductance which yields a different value according to the pressure variation (the input parameter). The sensitivity coefficient allows to know the effect on the measured output conductance due to a pressure change. The relation between the independent variable and the measured quantity is used as a calibration curve for developing the optimized sensor. The presence of an uncertainty in the conductance value during the dynamic mode operation gives information about the sensor reliability. The elastic behavior, the material of the fabric and the conductive formulation affect the sensor response after several working cycles. For example, the three-polyamide-layers (fabric A) and woven polyester fabrics (fabric C) are those that show the more stable response. The knitted-cotton based sensor (fabric D) is very unstable through the cycles and even if its sensitivity value calculated by analytical tools is comparable with that of polyester based sensors, it exhibits an excessive instability. In order to highlight the best materials and formulations for a real-life application of textile pressure sensors in a non-static framework, we computed the pressure uncertainty value δP_i_ in each single applied pressure as:

δPi = δGi∂G∂PP = Pi.

**Figure 7:**
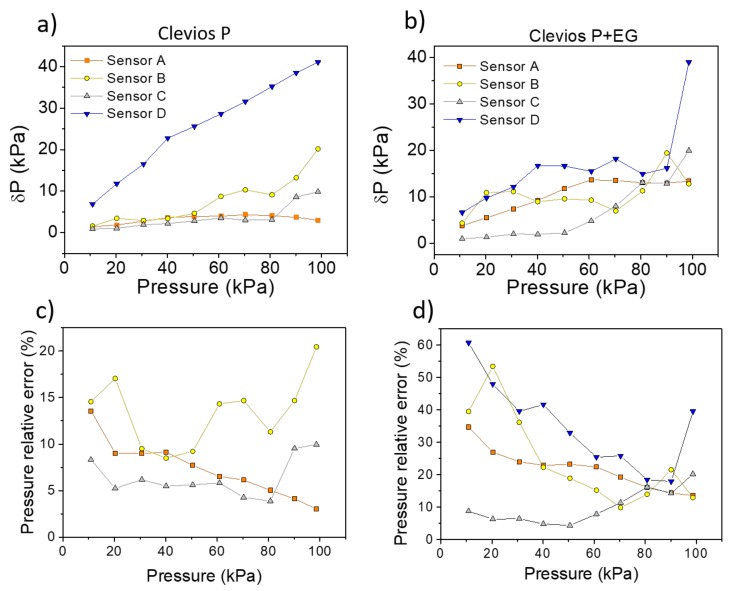
Pressure uncertainty values against the applied pressure (**a**,**b**). Here, for the sake of clarity, are reported the δP values only every 10 kPa. The inset in (**a)** also shows the trend of sensor D for the solution without ethylene glycol. The figures (**c**,**d**) report the pressure relative error for each sensor and for both formulations (excluding the sensor D with pristine formulation).

Using this formula, we take into account the single point sensitivity δGδPi calculated differentiating the curves in Figure 6, and the conductance uncertainty values $\delta G_{i}$ This equation allows the extraction of information about the pressure uncertainty even if the sensitivity coefficient is not constant over all the studied pressure range. Figure 7 reports the δP values for each sensor using both conductive formulations. The large instability in the dynamic operation with a subsequent huge uncertainty in the pressure estimation forced us to reject fabric D as a candidate for real pressure sensors. The presence of etylhene glycol also gives a similar instability, with an associated relative error ≥30%. A possible reason could be that cotton fibers coated with the conducting polymer increase their stiffness and this can lead to a delamination or to cracks during the compression and decompression cycles, resulting in a variable trend. Similar results have been reported in the literature for similar mechanical stress levels [32]. The relative error associated to sensor A with EG, indicates how it is not reliable when the second dopant is present in the solution, even if it has an average better performance than sensor B without EG. Finally, the sensor based on polyester fabric (C), even if it is poorly elastic (either in vertical and horizontal direction), is the more reliable one, both with and without ethylene glycol. Fabric C is therefore the best candidate among those considered by us in order to realize pressure sensors able to monitor and record a dynamic pressure variation.

## 4. Conclusions

This work investigates the physical mechanisms controlling the performance of textile pressure sensors directly fabricated onto different fabrics realized with natural and synthetic fibers. It presents a detailed study of how the fabric type, weaving and structure influences the pressure sensors performance in dynamic operation mode. It demonstrates how the mechanical properties of the fabric substrates affect the textile pressure sensor fabricated onto them.

We studied the mechanical and sensing features of textile based pressure sensors directly fabricated onto four different types of textile substrates such as polyamide (A and B), polyester (C) and cotton (D), and with different structures.

The elastic properties of the fabric used to realize the textile pressure sensor resulted in having a relevant impact on the sensor performance. Fabrics with good elastic properties and wide dynamic strain range during compression result in a highly sensitive sensor (fabric A and B).

However, also fabrics with poor elastic features can be used to realize good textile pressure sensors that, indeed under compression respond with a lower strain dynamic range and thus have a limited sensitivity, but guarantee a high reliability and stability in dynamic operation mode (fabric C).

Textile pressure sensor A, based on a three-knitted layer of polyamide, shows good performance and high sensitivity in an high dynamic range (30%) due to its elastic behavior. Sensors based on fabric B show a lower reproducibility for each cycle due to the intermediate elastic properties of the pristine fabric. In this case, the macroscopic geometrical change during compression and decompression affects the sensor performance in terms of reliability even if it presents the highest sensitivity value. Sensor C, based on polyester, is the fabric with the worst elastic properties and it shows a very good stability, except for the first compression cycle. After the first compression, the thickness of fabric C varies under stress with a very low dynamic strain range (10%) that, however, results in a reasonable electrical output signal. Its sensitivity is lower with respect to sensor A, but it can still be very useful for real life applications. Fabric D, the elastic cotton fabric, shows similar mechanical properties along the vertical direction to fabric C but sensors D and C exhibit a very different behavior. In the case of sensor D, it may be the microscopic level which affects the performance and, as suggested from the conductance plots in Figure 7, after each cycle delamination or cracks occurring in the coating may lead to a deterioration of the PEDOT:PSS on the surface [29]. We thus conclude that textile sensors, fabricated onto fabrics with good elastic properties in the vertical direction, indeed show a greater sensitivity but could be less reliable if compared to similar sensors deposited onto a stiffer fabric.

We also assessed the effects of varying the conductive polymer formulation. Textile pressure sensors realized onto four fabric substrates (A, B, C and D) with a PEDOT:PSS formulation containing ethylene glycol show on average a higher sensitivity, associated with a lower stability and lower reliability with respect to comparable pressure sensors fabricated onto identical fabric substrates but with a pristine PEDOT:PSS formulation.

In summary, the results we report in this work underline how to understand and validate the performance of textile pressure sensors in a dynamic operation mode. The sensitivity is not the only parameter that must be taken into account and the sensor’s response after multiple, repetitive compression cycles should also be considered. Fully textile pressure sensors are complex systems and in order to optimize them for real life applications it is important to consider several aspects, from the macroscopic to the microscopic scale. Reliability is an important and fundamental issue that should drive and guide the research and development of textile pressure sensors towards actual needs. We believe the results reported here open the way to a full optimization of textile pressure sensors in terms of most suitable fabrics, the range of linear pressure responses, sensitivity and dynamic operation behaviors to better fit the requirements of a large variety of dedicated real-life applications.

## Figures and Tables

**Table 1:** Summary of main parameters extracted from mechanical analyses.

	Creep Recovery				Stress-Strain Test
**Fabric**	**Initial Thickness (mm)**	**Thickness @100 kPa (mm)**	**Thickness after Release (mm)**	**Residual Deformation**	**Dynamic Strain Range**
A	2.49 *	1.34	2.36	5.3%	30%
B	1.54	0.74	1.40	8.5%	20%
C	0.36	0.19	0.29	20%	10%
D	0.71	0.37	0.57	20%	10%

* the DMA Q800 measures the thickness of the sample and the standard deviation associated to the average value correspond to a percentage error less than 1%.

**Table 2:** Main parameters of textile pressure sensors useful to evaluate their performances.

Clevios P			Clevios P + EG	
**Sensor**	**G_0_ (µS)**	**G_100_ (µS)**	**G_0_ (µS)**	**G_100_ (µS)**
A	0.09 ± 0.02	1.01 ± 0.02	2.1 ± 0.4	4.4 ± 0.2
B	1.4 ± 0.1	3.03 ± 0.09	1.1 ± 0.2	4.0 ± 0.4
C	0.314 ± 0.003	0.70 ± 0.01	0.35 ± 0.03	1.35 ± 0.07
D	0.05 ± 0.03	0.54 ± 0.07	0.05 ± 0.03	1.2 ± 0.1
	**S_FIT_ (nS/kPa)**	**Linear Range**	**S_FIT_ (nS/kPa)**	**Linear Range**
A	4.90 ± 0.04	60–100 kPa	16.2 ± 0.1	30–100 kPa
B	7.70 ± 0.07	50–100 kPa	25.6 ± 0.2	30–90 kPa
C	3.47 ± 0.03	40–80 kPa	8.3 ± 0.1	30–80 kPa
D	2.54 ± 0.03	40–100 kPa	7.9 ± 0.1	40–90 kPa

## Supplementary Materials

The following are available online at https://www.mdpi.com/1424-8220/19/21/4686/s1, Figure S1: Differential Scanning Calorimetry (DSC) thermogram pattern for the four fabric., Figure S”: DSC pattern for Fabric D (polyester) after washing, Figure S3: Compression cycles for textile pressure sensors realized with the addition in the conductive formulation of 10% *v*/*v* of ethylene glycol, Figure S4: Local sensitivity for each single sensor calculated differentiating the conductive curve, Figure S5. $\chi_{reduced}^{2}$ for the sensors based on Clevios P, Table S1: $\chi_{reduced}^{2}$ values for choose a linear operation range.
