# Supplementary material for: Impact of Fabric Properties on Textile Pressure Sensors Performance
Source: Sensors (Basel). 2019 Oct 28;19(21):4686. doi: 10.3390/s19214686 (PMC6864692; doi:10.3390/s19214686)
Supplement: Supplementary file 1 [file sensors-19-04686-s001.pdf]

Supplementary Materials:

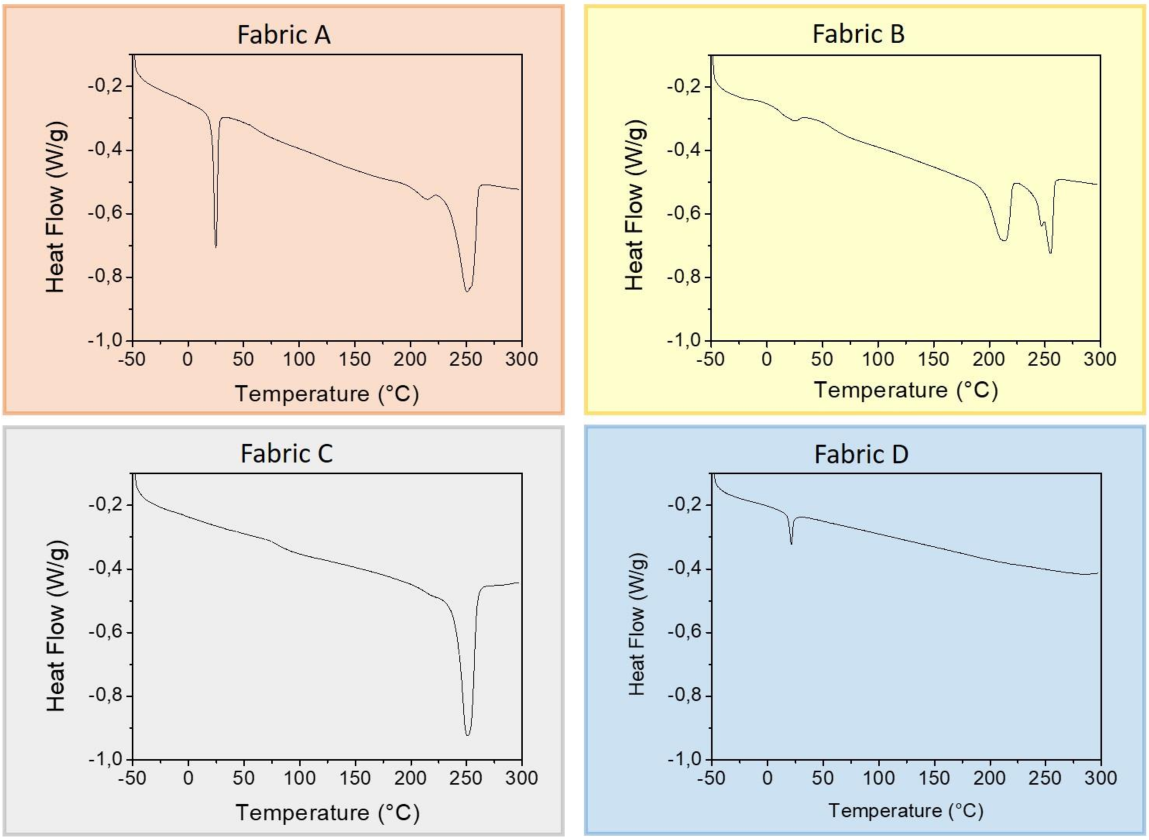

Figure SI1. Differential Scanning Calorimetry (DSC)thermogram pattern for the four fabric.

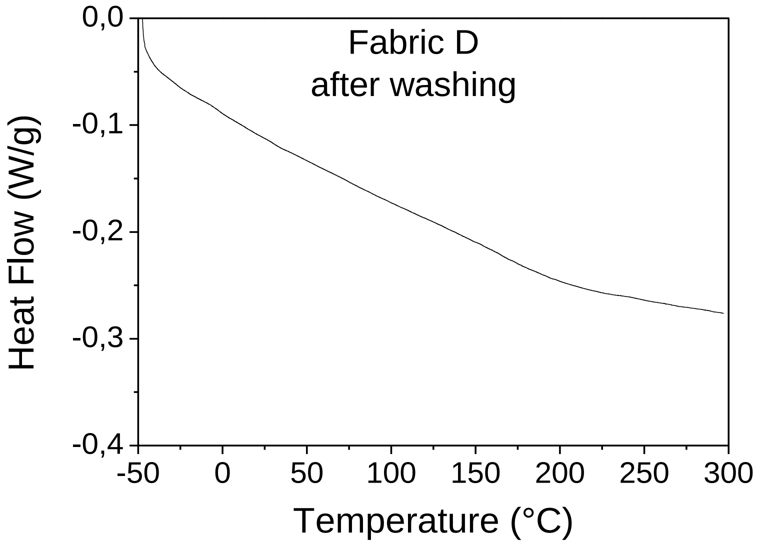

Figure SI2. DSC pattern for Fabric D (polyester) after washing.

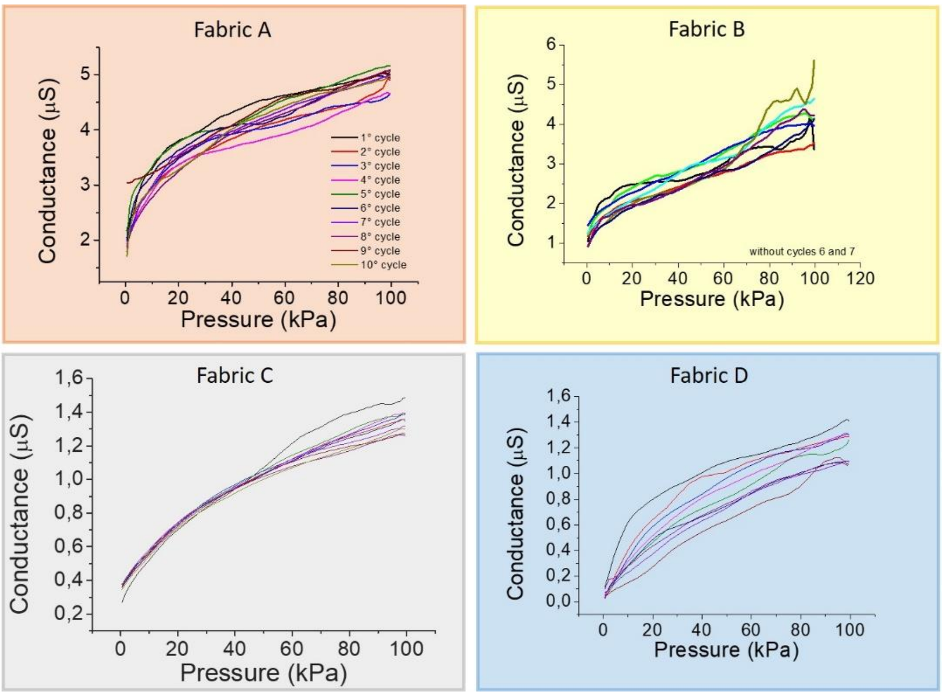

Figure SI3. Compression cycles for textile pressure sensors realized with the addition in the conductive formulation of 10% v/v of ethylene glycol.

Local Sensitivity

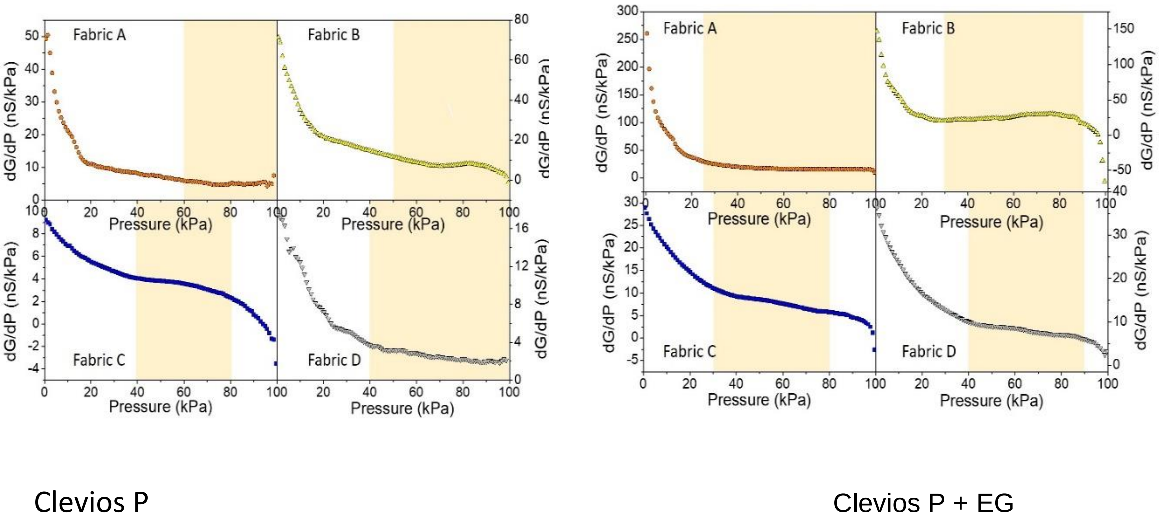

Figure SI4. Local sensitivity for each single sensor calculated differentiating the conductive curve.

Chi-square test

It is used to test the agreement between an observed data set and a certain expected behavior that came from a mathematical model. To find a region in which the trend of the conductivity is sufficiently linear with the applied pressure, we computed a weighed least square fit changing the X-range and we calculated for each different range the reduced χ^2 value (formula 1). Figure SI5 shows a common trend of the reduced χ^2 against the number of fitted points. The lowest is the reduced χ^2 values, the higest is the probability that the distribution used to explain the data set (in this case a linear behavior) is correct. Since the model takes into account the presence of the error in the y-value, the sensors that show a larger error σ_y have also a lower χ^2 value because the agreement with the proposed model is easier to reach. From these graphs, we extracted the linear operation range

applying a trade-off between the reduced χ^2 value, a reasonable range broadness and the constant region of the sensitivity plots in SI4.

$$\chi^2_{reduced} = \frac{1}{n-2}\sum_1^n \left(\frac{y_i - f(x_i)}{\sigma_i}\right)^2 \qquad (1)$$

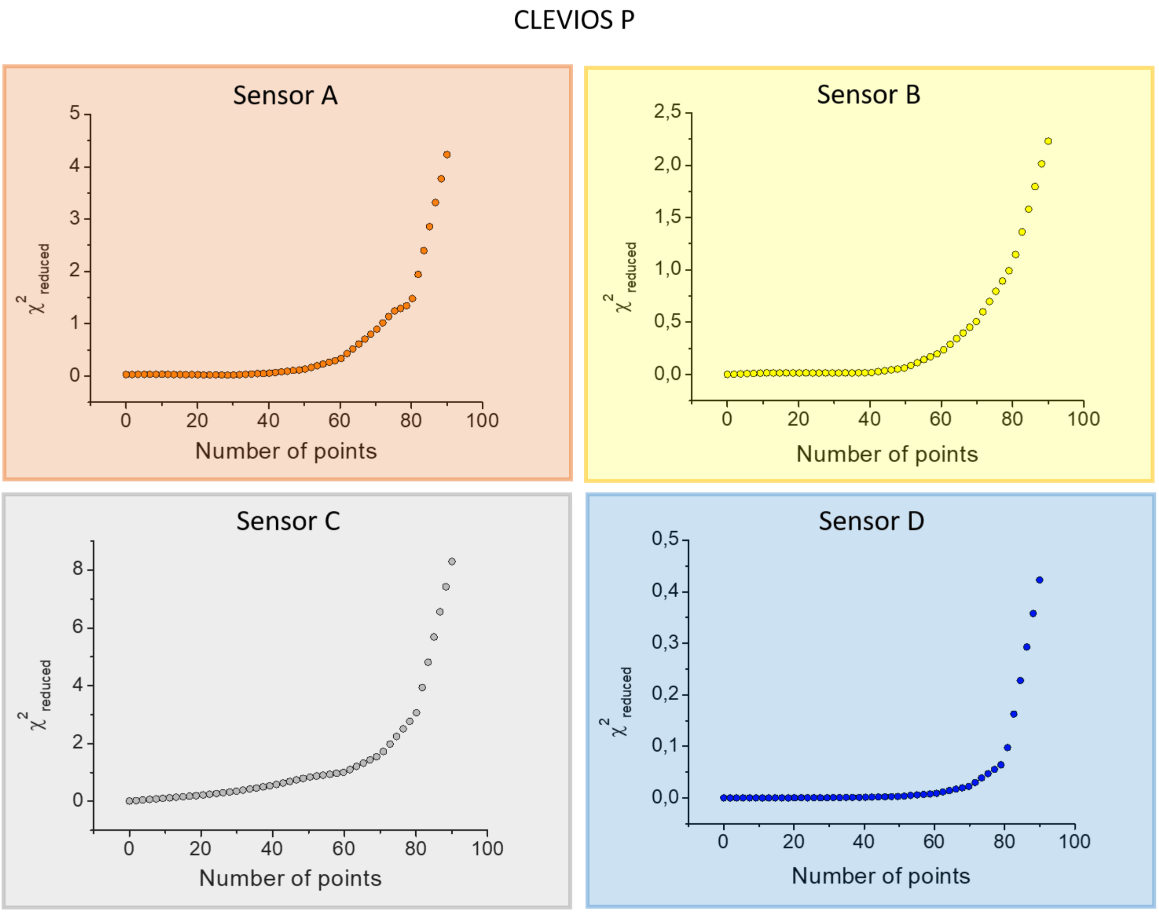

Figure SI5. $\chi^2_{reduced}$ for the sensors based on Clevios P. The number of points represent the number of data used to calculate the linear fit. This case is just an example of the $\chi^2_{reduced}$ distribution trend when the maximum independent value for the fit is 100 kPa. To find the best linear region, the highest pressure value has been changed to evaluate all possible combination.

Table SI1. $\chi^2_{reduced}$ values for choose a linear operation range.

$\chi^2_{Reduced}$	
**Clevios P**	**Clevios P + EG**
0,1	0,006
0,02	0,01
0,07	0,07
0,002	0,002
